# Long term functioning in early onset psychosis: Two years prospective follow-up study

**DOI:** 10.1186/1744-9081-7-28

**Published:** 2011-07-30

**Authors:** Ghada AM Hassan, Ghada RA Taha

**Affiliations:** 1 Institute of Psychiatry, Ain Shams University, WHO Collaborating Center for Training and Research, Abbasia, Cairo, Egypt

## Abstract

**Background:**

There were few studies on the outcome of schizophrenia in developing countries. Whether the outcome is similar to or different from developed world is still a point for research. The main aim of the current study was to know if patients with early onset non affective psychosis can behave and function properly after few years from start of the illness or not. Other aims included investigation of possible predictors and associated factors with remission and outcome.

**Method:**

The study prospectively investigated a group of 56 patients with onset of psychosis during childhood or adolescence. Diagnosis made according to DSM-IV criteria and included; schizophrenia, psychotic disorder not otherwise specified and acute psychosis. Severity of psychosis was measured by PANSS. Measures of the outcome included; remission criteria of Andreasen et al 2005, the children's global assessment scale and educational level.

**Results:**

Analysis of data was done for only 37 patients. Thirty patients diagnosed as schizophrenia and 7 with Psychotic disorder not otherwise specified. Mean duration of follow up was 38.4 +/- 16.9 months. At the end of the study, 6 patients (16.2%) had one episode, 23(62.1%) had multiple episodes and 8 (21.6%) continuous course. Nineteen patients (51.4%) achieved full remission, and only 11(29.7%) achieved their average educational level for their age. Twenty seven percent of the sample had good outcome and 24.3% had poor outcome. Factors associated with non remission and poor outcome included gradual onset, low IQ, poor premorbid adjustment, negative symptoms at onset of the illness and poor adherence to drugs. Moreover, there was tendency of negative symptoms at illness start to predict poor outcome.

**Conclusion:**

Some patients with early onset non affective psychosis can behave and function properly after few years from the start of the illness. Although remission is a difficult target in childhood psychosis, it is still achievable.

## Background

Schizophrenia is a serious impairment of higher brain functions including behavior, thinking, perception, emotions, and personality. It is one of the most disabling disorders as it was classified by the world bank as the fifth leading cause of years lost because of disability for men and sixth for women [1]. Although childhood schizophrenia is a rare disorder (approximately one in 10,000 children) [2], it may be more terrifying and debilitating condition for youth and family. Although the study of early-onset variants of disorders often enables the examination of a more genetically homogeneous and less environmentally influenced disease condition, there are little studies during childhood [3].

On the other hand, analyzing studies about the outcome of schizophrenia beginning in childhood or adolescence, one infers that the course and outcome is less favorable than in adult schizophrenic psychoses. Overall, it appears that schizophrenic adults were more likely to achieve periods of improvement, a higher level of psychosocial functioning and a better overall outcome [4-6].

Previous outcome studies of childhood schizophrenia tried to describe the course and types of outcome [4-7]. Moreover, studies investigated other predictors of outcome as the types of onset [8], duration of untreated psychosis [9], premorbid adjustment [5,6] and cognitive functions [3].

Reichert et al [4] investigated 27 former patients with childhood schizophrenia 13.4 years after first admission; they found that 77.8% of the former patients were still in outpatient treatment. Compared to the general population, the number of patients without a school graduation was relatively high (18.5%). Almost half of participants still live with their parents (48.1%) or in assisted or semi-assisted living conditions (33.3%). Only 18.5% were working in the open market. While Werry and his colleagues [7], investigated 30 former schizophrenic patients 7 to 17 years after their first inpatient treatment. Complete recovery was noted for only 23% of their patients at follow-up.

Hollis [10] reported that about one-fifth of patients in most studies have a good outcome with only mild impairment, while at the other extreme about one-third of patients are severely impaired requiring intensive social and psychiatric support. Limitations of previous studies included relative rarity of the disorder which reflected on the small sample size. Diagnosis of the disorder was another limitation as many cases diagnosed first as atypical or affective psychosis [11]. In addition, previous studies included conditions other than schizophrenia as schizoaffective disorder [6]. Other confounding factors were co-morbidities and past history with its consequences on outcome.

At the same time in developing countries especially Arab countries, there were little if any study regarding the outcome of childhood and adolescent onset psychosis. Moreover, there is no data to know whether the outcome of childhood schizophrenia was the same or different from studies done in developed countries.

So the main aim of the current study was to answer an important question which was "can patients with early onset psychosis behave and function properly after few years from start of the illness?" Other aims of the study included investigation of short term course, psychopathological and psychosocial outcome in a group of early onset non-affective psychosis. Also, this study was done to identify possible factors associated with good or poor outcome.

## Methods

### Design

This work was a longitudinal prospective study done in the period between January 2003 and June 2010.

### Setting

The study done at Al-Amal complex for mental health, Dammam, Kingdom of Saudi Arabia (KSA) after being approved by the scientific and ethical committee of the complex. A written informed consent was taken from all patients and/or their caregivers.

### Participants

Patients were recruited from the child psychiatry clinic according to certain inclusion and exclusion criteria. The patients' group included patients with non affective psychosis who got the disorder before age 18 years and did not pass the same age. In order to make the sample homogenous as much as possible exclusion criteria included any DSM-IV axis I diagnosis from the following disorders: schizoaffective, bipolar, major depression, substance induced psychotic disorder in addition to exclusion of cases with known organic pathology. However, patients with past history of epilepsy or febrile convulsions were not excluded.

### Timeline of the study

All patients presented to child and adolescence outpatient clinic were screened for psychosis through the general part of schedule for affective disorders and schizophrenia for school aged children present and lifetime version (K-SADS-PL) [12]. Ninety eight patients were positive for psychosis so assessed again in a second interview with the psychotic supplement of K-SADS-PL. Those who complete the required tools of the study and continued to follow up for minimal duration 24 months were 37 patients. Initial assessment was done two times by the two investigators who are child psychiatrists with good inter-rater reliability as Kappa was ≥ 0.8. Collection of statistically determined adequate sample was done in the period between 2003 and 2008.

Initial assessment was done at first and second visits to outpatient clinic (OPC) for all cases. The time between first and second visit was maximum one week. Follow up assessment was scheduled every 3 months all through the study. Minimum requirement for the study was one assessment every 6 months and follow up period of at least 2 years, otherwise considered as drop out.

Initial assessment included; 1) history taking and mental state examination, 2) K-SADS-PL for diagnosis and clinical assessment of psychotic symptoms [12], 3) Positive and negative symptoms scale (PANSS) for assessment of severity of psychosis [13], 4) IQ evaluation via Stanford Binet intelligence test (Arabic version [14], 5) Lewis Murray scale for assessment of obstetric complications [15], 6) Children's global assessment scale (CGAS) for evaluating the level of general functioning [16], 7) General developmental scale [6], 8) Child behavior scale (CBS)[17] and lastly 9) pre morbid adjustment scale (PAS) [18].

Assessment at follow up visits included; history taking and mental state examination, PANSS, the children's global assessment scale (CGAS), Andresen et al remission criteria [19].

At the last visit, function were assessed through three measures; Andresen et al remission criteria [19], global assessment scale and level of educational achievement.

### Assessment instruments

Many studies tried to answer the main study question but most of them chose sophisticated measures as neuropsychological testing and neuroimaging techniques [3,4,7]. Actually these measures are not used in routine clinical practice and in many developing countries there are no trained personnel to use such measures if available. That's why the investigators in the current study chose to assess behavior and brain functions of patients through more simple clinical bed side measures (children's global assessment scale, remission criteria of Andreasen et al [19] and level of education). These measures although simple, they are objective and have good sensitivity, specificity and reliability and can be used routinely and objectively to reflect the level of psychopathology.

The PANSS or the positive and negative syndrome scale [13] is a scale used for measuring symptom severity of patients with schizophrenia. The PANSS is a relatively brief interview, requiring 45 to 50 minutes to administer.

The children's global assessment scale (CGAS) [16], a measure of overall severity of disturbance, is an adaptation of the global assessment scale for adults. Findings indicate that the CGAS can be a useful measure of overall severity of disturbance. The scale has a numeric value (0 through 100) and used to rate the social, occupational, and psychological functioning of patients. Higher scores indicate better functions and were used in many studies to reflect the ability of the brain to perform and adapt to psychosocial functions.

GDS [6] was used to record early childhood developmental delay and neuro-developmental problems and covered seven areas: motor milestones, language milestones, social development, reading problems, neuro-developmental problems, enuresis and encorporesis. The total GDS score range from 0 to 12 and higher scores indicate more developmental impairments.

CBS [17] was used for rating of the premorbid period which ends 12 months before the onset of the first psychotic symptoms. The scale has 10 aspects: social isolation, social aloofness, separation anxiety; unusual stereotyped interest, deviant social communication, affect, suspiciousness, thought content, deviant speech and antisocial behavior. Total CBS Score is 0-20 and higher scores indicate more premorbid impairments.

PAS [18] is consisted of 28-items and assesses sociability and withdrawal, peer relationships, adaptation to school, and scholastic performance in four life stages (childhood, early adolescence, late adolescence; adulthood). Higher scores of PAS indicate poorer function. PAS also assesses socio sexual aspect after age 15 but this item was excluded from application because of cultural differences.

Remission was measured according to criteria of Andreasen et al [19]. Full remission is defined as achievement of remission on 8 symptoms from PANSS at any point of follow up and persistence for 6 months. These criteria were identified by factor analysis of PANSS and included 8 items which are delusions, unusual thought content, hallucinatory behavior, conceptual disorganization, mannerisms/posturing, blunted affect, social withdrawal and lack of spontaneity [19]. Partial remission was defined as presence of evidence of significant clinical improvement with less severe symptoms persisting (50% improvement from the baseline) [19,20].

Level of education was considered; average if the patient achieves his/her suspected educational grade, mild to moderate if there is delay 1-3 years less than same age colleagues, severe if there is delay more than 3 years.

The investigators put three outcome categories according to the best level of function (highest score of CGAS) in addition to remission status; Good out come if patients achieve remission and CGAS was ≥ 70. Moderate outcome if CGAS between 40-70 with full or partial remission. Poor outcome if CGAS ≤ 40 and partial or no remission.

Positive consanguinity was only considered in case of second degree consanguinity as defined by common law (uncle, Cousin). Similarly family history considered positive only in case of first and second degree (one parent, one sib or first cousin) [21].

Details about obstetric history and early development can be subjected to different kinds of bias such as recall bias or recency effect. In the current study we tried to minimize these effects through depending on retrieving the information from more than one source (family members, medical reports, etc) but definitely we didn't exclude bias completely and this may be the case in most of previous studies concerned with retrospective data.

Also the investigators used some operational definitions; Duration of illness was measured from onset of symptoms till last follow up in months. Duration of follow up was measured from first visit to clinic till the last visit within the study duration. Duration to improvement was measured from onset of symptoms till full remission or 50% response if no remission. Duration of untreated psychosis was measured from the onset of first psychotic symptoms to the start of treatment. Patients were considered adherent if missed doses were less than 25% of doses all through duration of follow up.

### Statistical analysis

Analysis of the data was done by using statistical program for social science (SPSS) version 10. The statistician chose suitable measures for the size of the sample to reduce attrition rate, mainly descriptive statistics, Chi square and cross stab for analysis of categorical variables. Mann Whitney and Kruskal-Wallis test were used to compare quantitative variables in the same group instead of independent group t-test and one way between groups ANOVA because of the non-parametric criteria of the data (SD > 50% mean). Spearman's rank correlation was also used. Logistic regression was used to detect possible predictor factors for remission and or outcome.

## Results

### Demographic and clinical characteristics

Out of 56 patients (26 males and 30 females) only 37 (14 males and 23 females) completed the minimum duration and follow up visits of the study. Mean age of patients 17 +/- 3.7, while mean age of onset of psychosis was 12.2 +/- 3.7. Thirty patients were diagnosed as schizophrenia (14 paranoid, 8 undifferentiated, 8 hebephrenic) and 7 with psychotic disorder not otherwise specified. The onset of illness was acute in 16 patients (43.2%) and gradual in 21 patients (56.8%). Mean IQ of the sample was 87.4 +/- 22 although 8 patients (21.6%) were in the category of mental retardation. In addition mean duration of illness was 61 +/- 39.9 months, mean duration of follow up was 38.4 +/- 16.9 months. Mean duration of untreated psychosis was 17.6 +/- 28 months.

### Diagnosis and Drop out

All findings regarding diagnoses and dropout can be found in table 1. Nineteen (33.9%) patients of the original sample didn't complete the study. Five patients were given different diagnoses during follow up (1 bipolar disorder, 2 schizoaffective and 2 substance induced psychosis) and 14 patients either didn't complete the minimum duration, visits and/or required tools. Comparing dropout to those who complete the study regarding, age of onset, sex, personal and family history, diagnosis, initial PANSS score and CGAS show no significant difference except for diagnosis as dropout was more among non schizophrenic group (P = 0.022).

**Table 1:** Diagnosis and dropout

Items		Number
Original sample size		56

Drop out		14

Exclusion due to change of diagnosis	Bipolar disorder	1
	
	Schizoaffective	2
	
	Substance induced psychosis	2

Schizophrenia at last visit		30

Schizophrenia at first visit		29

PDNOS at last visit		7

PDNOS at first visit		22

Acute psychotic episode at last visit		0

Acute psychotic episode at first visit		5

PDNOS: psychotic disorder not otherwise specified

In addition, table 1 gave an idea about diagnostic stability in the sample. Out of 5 patients with acute psychotic episode (APE) no one continued the study either due to drop out or change of diagnosis, while 7 patients out of 22 patients with psychotic disorder not otherwise specified (PDNOS) continued with the same diagnosis. Consequently number of schizophrenic increased from 29 to 30 patients at the end of the study.

### Past history and family history

Consanguineous marriage was found in 17 patients (45.9%) from the sample. In addition, 16 patients (43.2%) had positive family history of schizophrenia and 5 patients (13.5%) had family history of other psychiatric illness. Table 2) showed relation between family history and remission status and different groups of outcome, however there was no statistically significant difference. Furthermore, 17 patients (45.9%) had positive past history of psychiatric illness (7 mental retardation, 3 autism, 1 autism with mental retardation, 4 anxiety disorders and 2 conduct disorder).

**Table 2:** Showed relation between family history and remission status and different groups of outcome

FH	Remission	Non remission	Good outcome	Moderate outcome	Poor outcome
**Negative**	10 (62.5%)	6 (37.5%)	5 (31.3%)	8 (50%)	3 (18.7%)

**Schizophrenia**	7 (43.8%)	9 (56.3%)	5 (31.3%)	5 (31.3%)	6 (37.5%)

**Other**	1 (20%)	4 (80%)	0 (0%)	5(100%)	0 (0%)

**P**	.220		.098		

As shown in table 3, the age of onset of psychosis and its mode of onset were significantly related to early development and premorbid functions (GDS, CBS and PAS). Moreover, early development and premorbid functions (GDS, CBS, and PAS) were significantly related to psychotic symptoms (positive and negative) while current functioning (CGAS) was significantly related to negative symptoms and GPS.

**Table 3:** Correlation between premorbid state and PANSS, GAS, mode of onset and age of onset (r-value)

Premorbid function	PANSS			GAS	Mod of onset	Age of onset
	**Positive**	**Negative**	**GPS**			

**GDS**	-0.4**	0.2	-0.2	-0.09	**.2**	-0.5**

**CBS**	-0.3*	0.3*	-0.2	-0.1	.5**	-0.5**

**PAS**	-0.2	0.6**	-0.007	-0.3*	.4**	-0.5**

**CGAS**	-0.2	-0.4**	-0.4**	-----	-.1	-0.02

**Age of onset**	0.3*	-0.05	0.2	-0.02	-.3*	------

NB: * indicates significant p < 0.1; ** indicated highly significant p < 0.01

GDS: General developmental scale; CBS: Child behavior scale; PAS: Premorbid adjustment scale; GAS: Global assessment scale; GPS: General psychopathology scale.

As shown in table 3, more impairment in premorbid function as indicated by PAS is associated with more negative symptoms, which appear to affect the current functioning as shown by GAS. At the same time, the developmental and behavioral problems predating onset of psychosis (GDS, CBS) were significantly correlated to severity of psychotic symptoms, mode of onset and age of onset rather than current functioning (GAS).

### Outcome measures

At the end of the study 6 patients (16.2%) had only one episode, 11 patients (29.7%) had multiple episodes without deficit, 12 patients (32.4%) had multiple episodes with deficits and 8 patients (21.6%) had continuous course. Data regarding outcome measures are all summarized in table 4. Regarding remission; 19 patients (51.4%) achieved full remission, 9 patients (24.3%) had partial remission and 9 patients (24.3%) had no or little improvement. Mean duration till improvement (or remission) was 12.4 +/- 10.1 months, full remission achieved within 10.4 +/- 8.8 months. Regarding educational level at the end of follow up period; only 11 patients (29.7%) achieved their average educational level for their age while 15 patients (40.5%) had mild to moderate delay and 11 patients (29.7%) had severe delay. Although the policy of the hospital didn't allow admission before age 16 years, 10 patients (27%) of were grossly disturbed that necessitate admission.

**Table 4:** Showed outcome measures

Outcome measures	Items	Number (%)
**Remission**	Full remission	19 (51.4%)
	
	Partial remission	9 (24.3%)
	
	No remission	9 (24.3%)

**Educational level**	Average	11 (29.7%)
	
	Mild delay	15 (40.5%)
	
	Sever delay	11 (29.7%)

**Course**	Single - Episodic	15 (40.5%)
	
	Remission & exacerbation	13 (35.2%)
	
	Continuous	9 (24.3%)

**Outcome**	Good	10 (27%)
	
	Moderate	18 (48.6%
	
	Poor	9 (24.3%)

Comparisons between remittent and non remittent groups of patients found that there are many factors significantly affect the remission status as the diagnosis, onset of the illness and educational level. All these variables are presented in table 5. Comparison of remittent to non remittent patients showed statistically significant differences as remission was more with cases of acute onset of illness and good adherence to drugs. In addition, remission was more among patients of paranoid schizophrenia and PDNOS.

**Table 5:** Showed comparisons between remittent and non remittent groups

Items		Remittent Number (%)	Non remittent Number (%)	P
Sex	Male	6 (42.9)	8 (57.1)	0.42
		
	Female	13 (56.5%)	10 (43.5%)	

Diagnosis	Paranoid	10 (71.4%)	4 (28.6%)	0.006
		
	Undifferentiated	1 (12.5%)	7 (87.5%)	
		
	Disorganized	2 (25%)	6 (75%)	
		
	Atypical psychosis	6 (85.7%)	1 (14.3%)	

PH	Positive	6 (35%)	11 (65%)	0.296
		
	Negative	13 (65%)	7 (35%)	

FH	Schizophrenia	7 (43.8%)	9 (56.3%)	0.419
		
	Negative for schizophrenia	12 (57.1%)	9 (42.9%)	

Onset	Acute	12 (75%)	4 (25%)	0.012
		
	Gradual	7 (33.3%)	14 (66.7%)	

Compliance	Adherent	14 (70%)	6 (30%)	0.014
		
	Poor adherence	5 (29.4%)	12 (70.6%)	

Educational level	Average	10 (90.9%)	1 (9.1%)	0.006
		
	Mild delay	6 (40%)	9 (60%)	
		
	Severe delay	3 (27.3%)	8 (72.7%)	

PH: past history; FH: family history

P value is significant if ≤ 0.05; P value is highly significant if ≤ 0.005

Regarding categories of family history, there was no statistically significant difference between patients with positive family history and those with negative family history regarding remission status. Family history was positive for other psychiatric disorders only in 5 cases. Two cases of them were positive for mood disorders, one case was positive for mental retardation, one case for substance induced psychosis and the last case was positive for OCD.

Also comparisons between remittent and non remittent patients as regard continuous variables showed some significant differences as summarized in table 6. As remittent group had older age of onset and better premorbid functions, in addition to less negative symptoms and better IQ.

**Table 6:** Comparison between remittent and non-remittent regarding continuous variables:

Variables	Remittent	Non remittent	P value
**Age of onset**	13.5 +/- 3.3	11.1 +/- 3.7	0.03

**GDS**	1.5 +/- 2.5	3.4 +/- 3.3	0.05

**CBS**	4.9 +/- 4.7	8.3 +/- 3.9	0.02

**PAS**	9.7 +/- 5.2	14 +/- 4.9	0.01

**DUP**	9 +/- 9.8	27.9 +/- 43.9	0.092

**Duration to improvement**	10.4 +/- 8.8	21 +/- 20.3	0.092

**Positive subscale**	28.5 +/- 8.9	26.8 +/- 5.8	0.538

**Negative subscale**	25.5 +/- 11.3	34 +/- 10.1	0.024

**GPS**	56.8 +/- 12.2	54.7 +/- 16.1	0.408

**IQ**	93 +/- 25.1	82 +/- 17.5	0.04

GDS: general development scale; CBS: child behavior scale

PAS: premorbid adjustment scale; DUP: Duration of untreated psychosis

GPS: General psychopatrhology subscale from PANSS

IQ: Intelligent quotient; P value is significant if ≤ 0.05

Spearman correlation showed many factors significantly associated with remission as older age of onset (r = .354, P = .032) and less score on child behavior scale (CBS), general developmental scale (GDS) and premorbid adjustment scale (PAS) as (r = -.396 - P = .015), (r = -.327 P = .048) and (r = -.405 P = .013) respectively. Also, remission was associated significantly with higher IQ (r = .325, P = .050), less negative symptoms (r = -.375, P = .022) and compliance (r = -.355, P = 0.033). Moreover, shorter duration of untreated psychosis (DUP) and duration to improvement were significantly associated with remission state as (r = -.284 P = .088) and (r = -.328 P = .082) respectively.

### Regarding outcome

Also comparisons of groups of patients with different outcomes revealed some significant differences which are summarized in table 7. There were significant difference between groups as good outcome group had better PAS, less negative symptoms, less GPS score and better IQ. In addition good outcome were more in patients with PDNOS and paranoid schizophrenia rather than disorganized and undifferentiated schizophrenia.

**Table 7:** Comparison between groups with different outcomes regarding clinical and premorbid variables:

Variables	Good outcome	Moderate outcome	Poor outcome	P value
**Age of onset**	12.5 +/- 4.1	12.8 +/- 3.7	10.8 +/- 3.2	.255

**Sex**				
**Male**	3 (21.4%)	6 (42.9%)	5 (35.7%)	.445
	
**Female**	7 (30.4%)	12 (52.2%)	4 (17.4%)	

**GDS**	0.7 +/- 0.82	3.3 +/- 3.2	2.9 +/- 2.7	.165

**CBS**	4.7 +/- 5	7.5 +/- 4.7	7.1 +/- 3.1	.231

**PAS**	7.4 +/- 2.4	12.7 +/- 5.4	15.3 +/- 4.8	.002

**DUP**	12.1 +/- 10.5	20.2 +/- 42.6	23.2 +/- 29.7	.410

**Duration to improvement**	7.1 +/- 3.2	14.6 +/- 10.7	38 +/- 33	.082

**Positive subscale**	28.8 +/- 8.2	26 +/- 6.4	29.7 +/- 6.2	.343

**Negative subscale**	21.1 +/- 9.7	31.3 +/- 11	36.8 +/- 8.2	.009

**GPS**	57.7 +/- 12.7	50 +/- 12.4	65 +/- 14.8	.030

**IQ**	105.8 +/- 10.1	80.8 +/- 21.4	80 +/- 22.4	.008

**Diagnosis**				
**Paranoid**	5(35.7%)	7(50%)	2(14.3%)	.040
	
**Undifferentiated**	1(12.5%)	5(62.5%)	2(25%)	
	
**Disorganized**	0(0%)	3(37.5%)	5(62.5%)	
	
**PDNOS**	4(57.1%)	3(42.9%)	0(0%)	

GDS: general development scale; CBS: child behavior scale

PAS: premorbid adjustment scale; DUP: Duration of untreated psychosis

GPS: General psychopathology subscale from PANSS; IQ: Intelligent quotient

P value significant if ≤ 0.05; P value is highly significant if ≤ 0.005

PDNOS: Psychotic disorder not otherwise specified

Spearman correlation showed that many factors were significantly associated with good outcome as shorter duration to improvement (r = .419, P = .024), higher IQ (r = -.450, P = 0.005), less score on PAS (r = .595, P = 0.000), less negative symptoms (r = .506, P = 0.001), negative past history (r = .342, P = 0.038), acute onset (r = 349, P = 0.034) and good adherence to drugs (r = 339, P = 0.040).

One of the two factors constitutes the outcome in the current study was the remission status that's why there was highly significant differences between cases of single and remittent episodes in comparisons to cases of continuous course as shown in table 8.

**Table 8:** Showed relation between outcome and course

Course	Good outcome	Moderate outcome	Poor outcome	P
**Single/remittent**	10 (100%)	8 (44.4%)	0 (0%)	0.003
	
**Continuous**	0 (0%)	10 (55.6%)	9 (100%)	

Ten cases of single and remittent patients had good outcome and 8 cases had moderate outcome. While none of the cases with continuous course achieved good outcome. Moreover, the difference between the two groups of course as regard outcome was highly significant as P value was 0.013.

### Logistic regression

Binary logistic regression for possible predictors of remission revealed no significance as indicated by P = .814 (variables entered were PAS, Age of onset, negative symptoms and compliance). Also, binary logistic regression for outcome revealed no significance of the selected variables (PAS, negative symptoms and IQ). However, there was a tendency of negative symptoms to predict poor outcome as P value was 0.051.

## Discussion

The results of current study showed that 27% of patients with early onset psychosis can behave and function properly after few years from start of the illness as indicated by the remission of important brain and behavioral dysfunctions, good score on global assessment scale and average academic achievement in their education. Also, 48% of patients achieve moderate outcome and only 25% achieve poor outcome.

Regarding socio-demographic characteristics of the sample, those who complete the study were 37 patients, 14 males and 23 females. This discrepancy between number of females versus males was not statistically significance (df = 1 and P = .139). During follow up dropout was more in male group but still without statistical significance (df = 1, P = 0.075).

There was controversial findings regarding gender in previous studies as some studies reported male predominance [22,23] and some from the other studies showed female predominance [24,25]. Also, some studies found no gender difference [26]. Differences between studies may be the result of referral bias as reported by Hollis 's study [11].

Fifteen patients (40.5%) of the sample had only one episode, 13 patients (35.2%) had episodic course with no deficit and 9 patients (24.3%) had continuous course. During period of follow up 51.4% of the sample achieved remission while 24.3% showed partial remission and 24.3% showed little or no improvement. Comparing remittent to non remittent patients, remission was more among patients of psychotic disorder not otherwise specified and paranoid schizophrenia. Moreover, remission was more in patients with acute onset and good adherence to drugs.

Twenty seven percent of the sample (10 patients) had good outcome. This result was similar to Asarnow et al study [27] who reported a good level of psychosocial functioning in 28% of the cases.

The current study showed that 24.3% of the sample (9 patients) had poor outcome, while 45% of the sample of Asranow et al study [27] showed deteriorating course and minimal improvement.

Moreover, the results of the current study were better than that of Maziade and his colleagues [8], as only 5% of the sample in this study achieved full recovery after a mean follow-up interval of 14.8 years, with poor or very poor outcome in 74% of the sample.

Remschmidt et al study [28] examined the outcome of very early onset schizophrenia before age 14 years after a mean time span of 42 years and they found that 60% of patients had poor outcome and only 24% showed moderate global outcome.

These results were relatively better than study of Eggers and Bunk [24] who reassessed 44 inpatients after a long follow-up interval of 42 years and found only 25% of cases with full remission and another were 25% with partial remission and about 50% were poorly remitted.

Although there are a lot of methodological differences between the current study and those mentioned studies, it seems that the longer the duration of the study the more badly the outcome.

Another important aspects of psycho-social outcome investigated in previous studies were education, living conditions and occupational situation. In spite of short duration of the current study, 29.7% of the sample (n = 11) achieved their average educational level for their age. Similarly Remschmidt and his colleagues [28] reported that 74% of patients failed to graduate from any school and 71% were unemployed.

Comparing remission status to outcome status in the current study, 51.4% of the sample achieved remission but only 27% considered as good outcome group.

Relatively better remission rate and outcome in the current study than previous studies [8,24,27,28] may be due to shorter duration of follow up, different clinical characteristics of the sample, different outcome measures and remission criteria used. Use of social outcome measures and quality of life scales in those patients may reflect the poorest outcome measures.

Another possible explanation for better outcome in the current study is the environment in which the study was done. This study was done in a developing country and many studies found that outcome of adult schizophrenia is better in developing than developed countries [29,30]. So it may be more tolerance and acceptance of psychotic people and better family cohesion in developing countries rather than actual better global outcome of schizophrenia.

However recent data from worldwide-schizophrenia outpatient health outcomes (W-SOHO) study in 2009 reported that with a few exceptions, the W-SOHO study baseline findings generally show substantial similarity in outcome across countries representing various world regions outside of North America including 196 patients from Saudi Arabia [31].

### Possible predictors for remission and outcome

In this study, factors associated significantly with remission were older age of onset of psychosis, better premorbid functions (as measured by CBS, GDS, PAS and IQ), lesser negative symptoms at start of illness and more adherence to treatment. Although there were significant association between remission and previous factors however none of them could be considered as predictor for it, as logistic regression showed no significance.

In the current study, in spite of the high rate of positive consanguinity and positive family history we couldn't demonstrated the effect of this genetic load on outcome of our sample, these may be due to small sample size with different categories of family history and outcome.

On the other hand, factors associated with good outcome in the current study were good premorbid function (lower score of PAS), negative past history, higher IQ, acute onset, lesser negative symptoms, good adherence to drugs and shorter duration to improvement (Table 6). There was tendency of negative symptoms to predict poor outcome.

Similar associated factors or predictors were found in previous studies as age at onset of the disorder [2,24,32], the type of onset acute versus chronic [24,33], premorbid adjustment [6,7,34] and lower scores of negative symptoms [11]. Furthermore, previous studies reported IQ as strong predictor of social outcome [35].

Another possible predictor for remission first investigated by Hollis [36] was early course over the first 6 months or as defined in the current study "time to improvement".

Although DUP was one of the possible predictors in some previous studies of adult onset psychosis, it was not the case in the current study. This finding may be similar to Beng-Choon et al [9] and it means that duration of untreated initial psychosis is not a prognostic factor for outcome early in the course of schizophrenia, however this point still in need for further analysis and research.

Furthermore, some cases in the current study had concomitant intellectual disability or autism and this can certainly affect the clinical outcomes and longitudinal progress. However, schizophrenia is a neurodevelopmental disorder and premorbid symptoms are common especially in early onset cases. Previous studies similarly included patients with positive past history [32,37]. Regarding MR, several studies have found a mean IQ between 80 and 85 (one standard deviation below the population mean), with about one-third of cases having an IQ below 70 [36,38,39]. So some authors consider low IQ as one of the vulnerability factors for schizophrenia and others consider it as an expression of impaired early brain development [40]. For sure these comorbidities impact the outcome of psychosis but clinically cleaning up of the sample and removal of such impacts is extremely difficult in naturalistic prospective studies. Moreover, sample heterogeneity and comorbidities give readers more chance to generalize the results to all cases of early onset non affective psychosis.

### Strengths and limitations

In spite of the fact that, low or middle-income countries represent more than 85% of the world's population, only 6% of publications come from it [41]. Prospective longitudinal research in Middle East countries is minimal if any because of many logistic problems. Moreover, research in the field of outcome of early onset psychosis is scanty. That's why the current study is a unique prospective naturalistic study of childhood and adolescent onset psychosis. Prospective design of the study allowed the comment on course and inter-episodic periods and overcome gaps in medical recording of files and registration bias of retrospective studies. In addition the investigators used a lot of valid tools in initial assessment and all through follow up (K-SAD PL, PAS, PANSS and CGAS) to strengthen the results. Also, Initial assessment and clinical tools were done by 2 child psychiatrists with good inter rater reliability. Moreover, the current study focused on previously discussed predictive factors for outcome together with emphasis on possible new outcome measures as time to improvement and compliance.

However due to prospective design there was high rate of drop out (33.9%) from the original sample. Furthermore, in comparisons to other outcome studies [4,7,28] the mean duration of illness and follow up in the current study was shorter (61 +/- 39.9 and 38.4 +/- 16.9 months respectively). That's why this work was an intermediate term outcome study.

Diagnostic heterogeneity within the subject weakened the power and focus of the study but on the other side this heterogeneity may be considered a strong point for two reasons. The first, the subjects here were a naturalistic sample from real life clinical practice and gave an idea about the outcome in all cases of early onset non affective psychosis whatever the system of classification used and whatever the heterogeneity or comorbidity. The second reason, psychosis is a continuum and the demarcation between cases currently used in psychiatry is a bias because of its dependence on categorical classification without any relation to any biological etiology. Moreover, many of previous studies included other diagnoses [3,4,6,17,36]

## Conclusion

Remission and good outcome of behavioral and brain dysfunctions of patients with early onset non affective psychosis is attainable. There are some factors that consistently related to remission and good outcome which were acute onset, higher IQ, good premorbid adjustment, less negative symptoms at start of the illness and good adherence to medications. In addition, there is tendency of negative symptoms at illness start to predict outcome. Also, the study put a question mark on the role of DUP on remission and outcome with new emphasis on the role of duration to improvement. However, screening of high risk children as those with low IQ, poor premorbid adjustment and positive family history for psychosis might help in early detection. Optimum intervention and strategies to improve compliance and negative symptoms are key points in both remission and good outcome. Although remission is a difficult target in childhood psychosis, it is still achievable.

## Competing interests

The authors declare that they have no competing interests.

## Authors' contributions

Both authors conceived of the study and participated in its design and coordination. GAMH administered the instruments and collected the data. GRAT participated in data collection and administration of tools. Both authors participated in the writing and revision and approved the final manuscript.
